# SOCIAL ENGAGEMENT IN OLD AGE: IMPACTS OF AGING UNDER INTERPERSONAL, MORAL, AND POLITICAL CONTEXTS

**DOI:** 10.1093/geroni/igz038.2886

**Published:** 2019-11-08

**Authors:** Minjie Lu, Xin Zhang

**Affiliations:** 1 Sun Yat-Sen University, Guangzhou, Guangdong, China; 2 Peking University, Beijing, China

## Abstract

To promote successful aging, social engagement has been encouraged among older adults. Yet, thus far, research on older adults’ social engagement have been preoccupied with the close and intimate relationships between older adults and their families, friends, or caretakers. Little attention is being paid to how older adults may engage in peripheral social networks, civic activities, or public affairs. This symposium features four presentations that investigate older adults’ social engagement in these contexts. First, with the focus on interpersonal interaction with strangers, Yi Lu and colleagues examine the age differences in trustworthiness perception of unfamiliar faces. The second and third presentations focus on moral and political contexts. Minjie Lu will present the divergent impacts of age on the cognitive evaluations and emotional responses towards moral issues. Wong and Fung analyze older adults’ engagements in political discussions and actions and the factors that may promote these engagements. Last but not least, Nicole Fung and colleagues will present findings that generativity among older adults can promote sense of meanings and death acceptance, demonstrating the beneficial effects of social engagement in old age. Finally, Zhang will provide closing discussions about the implications and future directions of these presentations.

